# *Bulletin* comment: What is wrong with the likes of Rolf Harris?

**DOI:** 10.1192/pb.bp.114.049718

**Published:** 2015-06

The revelations about the perverse activities of high-profile celebrities such as Rolf Harris, Jimmy Savile and Max Clifford continue to cause shock, bewilderment and voyeuristic intrigue. It seems the scourge of abuse is so prevalent that it extends into all institutions previously held in high regard; from the county councils, to the Catholic Church, and further to the Houses of Parliament.

For mental health professionals (as well as any other keen observer of human behaviours), such disturbing disclosures often lead to discussions about why any individual might act this way: considerations of personality disorders may be had. The extreme arrogance, the lack of regret and absence of profound feelings of guilt, in the context of lifelong careers in the media or entertainment industries or positions of importance in society, point towards cluster B difficulties, with elements of both antisocial and histrionic disorders.

News reports inevitably pay most attention to the wrongdoer and the calls for enquiries into the institutions, organisations or official bodies that have essentially been supportive or at least responsible for passively allowing the abuse to continue.

Ultimately, the revelations lead to thoughts about the trail of destruction left in the wake of such behaviours. Extensive research has repeatedly linked child sexual abuse with mental health difficulties of later life. Severe depression, anxiety problems, post-traumatic stress disorder and long-term inability to adjust to adult life: some studies have found life prevalence rates of up to 80% in victims of sexual abuse.

Although the media focus is on the activities of celebrities or society leaders, they are clearly responsible for only a tiny fraction of all childhood and adult sexual abuse cases. This high prevalence in the community setting places the problem fairly and squarely in the realm of public health. And as such, it should be the focus of effective education programmes and community-based treatments for sex offenders. The instruction of children and adolescents on human sexuality and the importance of interpersonal skills needed to build satisfying and healthy intimate relationships needs to be emphasised and funded.

Incarcerating the likes of Rolf Harris might satisfy the public’s desire for justice or their need for prurient stimulation, but in reality it does little for primary prevention of a continuing societal scourge.

